# Relative Frequencies of Alloantigen-Specific Helper CD4 T Cells and B Cells Determine Mode of Antibody-Mediated Allograft Rejection

**DOI:** 10.3389/fimmu.2018.03039

**Published:** 2019-01-22

**Authors:** Jawaher Alsughayyir, Manu Chhabra, M. Saeed Qureshi, Mekhola Mallik, Jason M. Ali, Ivonne Gamper, Ellen L. Moseley, Sarah Peacock, Vasilis Kosmoliaptsis, Martin J. Goddard, Michelle A. Linterman, Reza Motallebzadeh, Gavin J. Pettigrew

**Affiliations:** ^1^School of Clinical Medicine, University of Cambridge, Cambridge, United Kingdom; ^2^Department of Pathology, Papworth Hospital, Papworth Everard, United Kingdom; ^3^Histocompatibility and Immunogenetics Laboratory, Cambridge University Hospitals NHS Foundation Trust, Cambridge, United Kingdom; ^4^Laboratory of Lymphocyte Signalling and Development, Babraham Institute, Cambridge, United Kingdom; ^5^Division of Surgery and Interventional Sciences, University College London, London, United Kingdom; ^6^Centre for Transplantation, Department of Renal Medicine, University College London, London, United Kingdom; ^7^Institute of Immunity and Transplantation, University College London, London, United Kingdom

**Keywords:** allograft, humoral alloimmunity, germinal center (GC), extrafollicular B cell response, transplantation, vasculopathy

## Abstract

Humoral alloimmunity is now recognized as a major determinant of transplant outcome. MHC glycoprotein is considered a typical T-dependent antigen, but the nature of the T cell alloresponse that underpins alloantibody generation remains poorly understood. Here, we examine how the relative frequencies of alloantigen-specific B cells and helper CD4 T cells influence the humoral alloimmune response and how this relates to antibody-mediated rejection (AMR). An MHC-mismatched murine model of cardiac AMR was developed, in which T cell help for alloantibody responses in T cell deficient (*Tcrbd*^−/−^) C57BL/6 recipients against donor H-2K^d^ MHC class I alloantigen was provided by adoptively transferred “TCR75” CD4 T cells that recognize processed H-2K^d^ allopeptide via the indirect-pathway. Transfer of large numbers (5 × 10^5^) of TCR75 CD4 T cells was associated with rapid development of robust class-switched anti-H-2K^d^ humoral alloimmunity and BALB/c heart grafts were rejected promptly (MST 9 days). Grafts were not rejected in T and B cell deficient *Rag2*^−/−^ recipients that were reconstituted with TCR75 CD4 T cells or in control (non-reconstituted) *Tcrbd*^−/−^ recipients, suggesting that the transferred TCR75 CD4 T cells were mediating graft rejection principally by providing help for effector alloantibody responses. In support, acutely rejecting BALB/c heart grafts exhibited hallmark features of acute AMR, with widespread complement C4d deposition, whereas cellular rejection was not evident. In addition, passive transfer of immune serum from rejecting mice to *Rag2*^−/−^ recipients resulted in eventual BALB/c heart allograft rejection (MST 20 days). Despite being long-lived, the alloantibody responses observed at rejection of the BALB/c heart grafts were predominantly generated by extrafollicular foci: splenic germinal center (GC) activity had not yet developed; IgG secreting cells were confined to the splenic red pulp and bridging channels; and, most convincingly, rapid graft rejection still occurred when recipients were reconstituted with similar numbers of *Sh2d1a*^−/−^ TCR75 CD4 T cells that are genetically incapable of providing T follicular helper cell function for generating GC alloimmunity. Similarly, alloantibody responses generated in *Tcrbd*^−/−^ recipients reconstituted with smaller number of wild-type TCR75 CD4 T cells (10^3^), although long-lasting, did not have a discernible extrafollicular component, and grafts were rejected much more slowly (MST 50 days). By modeling antibody responses to Hen Egg Lysozyme protein, we confirm that a high ratio of antigen-specific helper T cells to B cells favors development of the extrafollicular response, whereas GC activity is favored by a relatively high ratio of B cells. In summary, a relative abundance of helper CD4 T cells favors development of strong extrafollicular alloantibody responses that mediate acute humoral rejection, without requirement for GC activity.

This work is composed of two parts, of which this is Part I. Please read also Part II: Chhabra et al., 2019.

## Introduction

The *de novo* development of alloantibody against donor MHC antigen following solid organ transplantation is now recognized as a major determinant of transplant outcome (1–5). For example, alloantibody formed within the first year of transplantation is associated with significantly poorer heart graft survival (6). In general, damaging alloantibody responses are associated with two distinct clinico-pathological processes: acute and chronic antibody mediated rejection (AMR). Acute AMR is now characterized for all solid organ transplants [reviewed in (7, 8)], whereas chronic AMR has been recognized only relatively recently (9), and remains ill-defined for some organs (10). Acute AMR affects 5–7% of non-sensitized kidney transplant recipients, is generally associated with high levels of Ig-switched alloantibody directed against mismatched MHC class I and/or class II antigens, and usually occurs within the first 6 months after transplantation. Treatment, typically with plasmapheresis and intravenous immunoglobulin, is less successful than following treatment for acute cellular rejection, and acute AMR is associated with an ~5-fold greater risk of graft loss at 5 years (11).

The link between different clinical manifestations of AMR and the causative cellular events in the allospecific B cell population is not clear. Alloantibody production is a typical T-dependent response, with help for allospecific B cells provided by “indirect-pathway” CD4 T cells that recognize target MHC alloantigen as self-restricted processed allopeptide (12, 13). Following B cell receptor (BCR) ligation, allospecific B cells would be expected to migrate in lymphoid tissue to the edges of the B cell follicle, and, upon productive “cognate” interaction with the indirect-pathway helper CD4 T cell, further differentiate along one of two, mutually exclusive pathways. In the extrafollicular response, help provided by CD44^hi^ICOS^hi^PSGL-1^lo^Bcl-6^+ve^ CD4 T cells (14–16), enables the B cell to migrate to short-lived foci within the red pulp in the spleen and medullary cords of lymph nodes for rapid production of low-affinity antibody (17). In contrast, B cell migration back to the follicle triggers a germinal center (GC) response, with development of the classical secondary follicle composed of a light and dark zone. The GC response is now known to be dependent upon a specialized subset of CXCR5^hi^ PD-1^hi^ T follicular helper (T_FH_) cells (18, 19).

While the extrafollicular and GC components of the response to model antigens have been extensively studied (20–22), they have not been detailed for transplant antigen. This is an important area for further study, because of the importance of humoral immunity to transplant rejection, and because transplantation provides a functional readout (graft rejection), that by enabling assessment of the “effectiveness” of the various components of the humoral response, may reveal aspects of humoral immunity that are not otherwise evident from study of model antigen systems. Equally, transplantation represents a unique immune challenge, in that vascularized allografts may continually shed alloantigen directly into the recipient's circulation and T cell recognition of this alloantigen can occur by different pathways (23–25). The relationships between the precursor populations of allospecific helper T cells to B cells may therefore differ for different donor-recipient combinations, and these differences may independently influence the subsequent extrafollicular and GC alloantibody responses. This may be particularly relevant for transplant recipients with acute AMR related to *de novo* production of donor-specific alloantibody. It seems likely that graft injury is mediated predominantly by an extrafollicular response, particularly during the initial stages. Certain patients may therefore be especially susceptible to early humoral rejection. However, the factors that determine the relative “strength” of the extrafollicular and GC alloantibody responses remain unclear, as does the respective contribution of the two phases to acute AMR.

Here we use murine models of AMR to demonstrate that a high ratio of antigen-specific helper CD4 T cells favors development of robust extrafollicular responses, and that these responses can mediate acute AMR without requirement for a GC component.

## Materials and Methods

### Animals

C57BL/6 (BL/6; H-2^b^) and BALB/c mice (H-2^d^) were purchased from Charles River Laboratories (Margate, UK) and maintained according to the institutional guidelines of The University of Cambridge. T cell receptor-deficient mice (H-2^b^, *Tcrbd*^−/−^) BL/6.129P2-*Tcrb*^*tm*1*Mom*^*Tcrd*^*tm*1*Mom*^/J were purchased from the Jackson Laboratory (Bar Harbor, ME). C57BL/6 *Rag2*^−/−^ mice (H-2b) were gifted by Prof T. Rabbitts (Laboratory of Molecular Biology, Cambridge, UK). TCR-transgenic *Rag1*^−/−^ TCR75 mice (H-2^b^), specific for I-A^b^-restricted H-2K${}_{54-68}^{\text{d}}$ peptide (26) were gifted by Prof. P. Bucy (University of Alabama, Birmingham, AL). BL/6 HEL-specific TCR7 transgenic mice (27), specific for I-A^b^-restricted HEL_74−88_ peptide, were gifted by Dr M Linterman (Laboratory of Lymphocyte Signaling and Development, Babraham Institute, Cambridge, UK). *Sh2d1a*^−/−^ mice (28) were gifted by Dr S. Crotty (University of California, La Jolla, California). Mice were bred and maintained in specific pathogen-free animal facilities and were maintained in individual ventilated cages in specific-pathogen free facilities and fed standard rodent feeds. Mice weighed between 18–22g at the time of their use for *in vitro* experiments and transplants. See also Table S1.

### Heterotopic Cardiac Transplantation

Vascularized cardiac allografts were transplanted intra-abdominally as previously described (24, 29). Recipient BL/6 *Tcrbd*^−/−^ or *Rag2*^−/−^ animals were reconstituted with TCR75 CD4 T cells, by intravenous injection of splenocytes from *Rag1*^−/−^ TCR75 mice in which numbers of CD4 T cells were first determined by flow cytometry. Hearts or cells from male donors were not transplanted or transferred into female animals in order to control for mismatch of the male H-Y antigen. Heart graft survival was monitored by daily abdominal palpation with rejection defined as cessation of a detectable beat, and rejection was defined as cessation of palpable myocardial contraction, confirmed at the time of explant. Grafts were excised at predetermined time points after transplantation and stored at −80°C or fixed in 10% buffered formalin.

### Immunizations and Adoptive Cell Transfers

BL/6 *Tcrbd*^−/−^ were immunized subcutaneously with 50 μg purified HEL protein (Sigma-Aldrich Inc., St. Louis, MO, USA) emulsified in complete Freund's adjuvant (Sigma-Aldrich). Immunized mice were subsequently adoptively transferred with prerequisite numbers of HEL-specific B cells and CD4 T cells that were first isolated from spleens of donor SW_HEL_ and TCR7 mice, respectively, and then enumerated by flow cytometry and Trucount^TM^ (BD Biosciences, San Jose, CA) analysis by staining with the following antibodies: CD3-FITC, CD19-PE (both BD Biosciences), and HEL protein conjugated with biotin (developed in-house) followed by Streptavidin-Alexa Fluor 555 (Thermo Fisher Scientific, Waltham, MA, USA) for HEL^+^ B cells, and CD4-APC with Vβ3-PE (both BD Biosciences) for HEL-specific CD4 T cells.

### Passive Immune Serum Transfer

In certain experiments, *Rag2*^−/−^ recipients of BALB/c heart allografts were intra-peritoneally injected with heat-inactivated pooled immune serum (500 μl 3 times weekly for 3 weeks or for shorter duration if the heart allografts had rejected), obtained at 50 days after transplantation from BL/6 *Tcrbd*^−/−^ recipients of a BALB/c heart allograft reconstituted with 5 × 10^5^ *Rag1*^−/−^ TCR75 CD4 T cells.

### Assays of Anti-H-2K^d^ and Anti-HEL Humoral Immunity

Serum samples were collected from experimental mice at intervals and analyzed for the presence of anti–H-2K^d^ and anti-HEL IgG antibody by ELISA as previously described (12, 13, 30) (Figure S1). For each sample, an absorbance vs. dilution curve was plotted, and the area under the curve calculated (31) and expressed as the percentage of positive control (pooled hyperimmune anti-H-2K^d^ IgG) serum recovered from BL/6 recipients of BALB/c skin grafts (Figure S1) or HyHEL10 anti-HEL IgG monoclonal antibody (Absolute Antibody, Oxford, UK). Single anti-H-2K^d^ or anti-HEL IgG antibody-secreting cells in the spleen and bone marrow of recipient mice were detected by B cell ELISPOT assay as previously described (13, 32).

### Recipient Anti-HLA-specific Antibody Screening

Non-sensitized recipients who received a primary deceased donor kidney transplant at Cambridge University Hospital were monitored for the development of HLA antibodies using the Luminex-based LABScreen Single Antigen beads (One Lambda, Canoga Park, CA) as described previously (33). As in our previous studies, IgG MFI values more than or equal to 1,500 were considered positive (34).

### CFSE Cell Proliferation and Flow Cytometry

Suspensions of CD4 T cells obtained from TCR75 mice were stained with 5 mM CFSE (Thermo Fisher Scientific) in the dark for 5 min and then quenched with PBS plus 5% FCS. CFSE-stained CD4 T cells were injected intravenously into recipient mice, spleens harvested 3 days later, and flow cytometry performed using allophycocyanin-conjugated anti-CD4 plus PE-conjugated anti-CD90.1/Thy1.1 (clone OX-7, BioLegend, San Diego, CA, USA) to identify live (7-AAD^−^, BD Biosciences) TCR75 T cells. PE-conjugated anti-mouse CD279/PD1 (BioLegend) and allophycocyanin-conjugated anti-mouse CXCR5 (BD Biosciences) antibodies were used to identify T_FH_ cells on a FACSCanto II flow cytometer (BD Biosciences).

### Immunohistology and Confocal Imaging

Seven μm spleen and heart cryostat sections were air-dried and fixed in acetone. Primary mAbs specific for the following mouse epitopes were used for immunohistochemical/fluorescent staining: C4d (clone 16-D2 Abcam, Cambridge, UK) and IgG-FITC (BD Biosciences, San Diego, CA, USA). Splenic GCs were identified by double-labeling sections with rat anti-mouse B220-APC (clone RA3-6B2) and rat anti mouse GL7-FITC (both BD Biosciences). Numbers of GL7^+^ GCs were expressed as a percentage of total B220^+^ lymphoid follicles (13). HEL-specific GCs were detected using rat anti-mouse GL7-FITC and biotinylated-HEL protein (developed in-house) combined with Streptavidin-Alexa Fluor 555 (Thermo Fisher Scientific) after initially blocking endogenous avidin/biotin activity using an avidin/biotin blocking kit (Vector Laboratories, Burlingame, CA, USA). CD4 T cells within GCs were located with rat anti-mouse CD4-biotin (BD Biosciences) & Streptavidin-Alexa Fluor 555 (Thermo Fisher Scientific). Confocal images were captured with a Leica SP5 confocal microscope using LAS AF software, version 2.7.2.9586 (Leica Microsystems, Wetzlar, Germany). See also Table S1.

### Flow Cytometric Detection of Alloantigen and HEL-Specific B Cells

Alloantigen-specific B cells were identified by labeling with synthetic MHC class I tetramer as described previously (35–37). Single cell suspensions were obtained from recipient spleens and incubated with APC- and FITC-conjugated MHC class I K^d^ tetramers, kindly gifted by the NIH Core Tetramer facility, Atlanta, GA, USA. B cells binding to fluorescently labeled tetramers can comprise of two distinct populations of B cells—those recognizing the MHC class I molecule directly or those recognizing the fluorochrome; dual labeling allows for reliable identification of very low frequency antigen-specific B cells. After 1 h incubation at 4°C, cells were washed and tetramer-bound cells were enriched using anti-APC and anti-FITC microbeads (Miltenyi Biotec) on an Automacs^TM^ separator. The enriched fraction was collected and cells were incubated with anti-mouse CD16/CD32 (clone 2.4G2) before labeling for the following antigens: CD19-PerCp (Miltenyi Biotec), GL7-PE (BioLegend), FAS-PE-CY7 (Jo2, BD Biosciences), and fixable viability dye (eFluor^®^ 780, eBiosciences). Labeled cells were identified on a FACSCanto II flow cytometer and data analyzed using FLowJo software (Tree Star Inc., Ashland, OR). B cell populations of interest were enumerated by Trucount^TM^ analysis according to manufacturer's instructions (BD Biosciences). HEL-specific GC B cells were identified in similar fashion, by labeling splenic single cell suspensions with B220-FITC (BD Biosciences), biotinylated-HEL protein and Streptavidin-APC (Thermo Fisher Scientific).

### Statistical Analysis

Data were presented as mean ± S.E.M where appropriate, with each animal constituting one biological replicate where indicated. Unpaired *t-*tests and Mann-Whitney *U*-tests were used for analysis of parametric data and non-parametric data, respectively. Two-way ANOVA was employed for comparison of anti-H-2K^d^ IgG alloantibody responses. Graft survival was depicted using Kaplan-Meier analysis and groups compared by log-rank (Mantel-Cox) testing. Analysis was conducted using GraphPad 4 (Graph-Pad Software, San Diego, CA, USA). Values of *P* < 0.05 were considered significant.

### Study Approval

This research has been regulated under the Animals (Scientific Procedures) Act 1986 Amendment Regulations 2012 following ethical review by the University of Cambridge Animal Welfare and Ethical Review Body (AWERB). All surgery was performed under inhalational anesthesia and every effort was made to minimize suffering.

## Results

### Post-transplant HLA Antibodies in Human Renal Transplant Recipients

**Figure 1:**
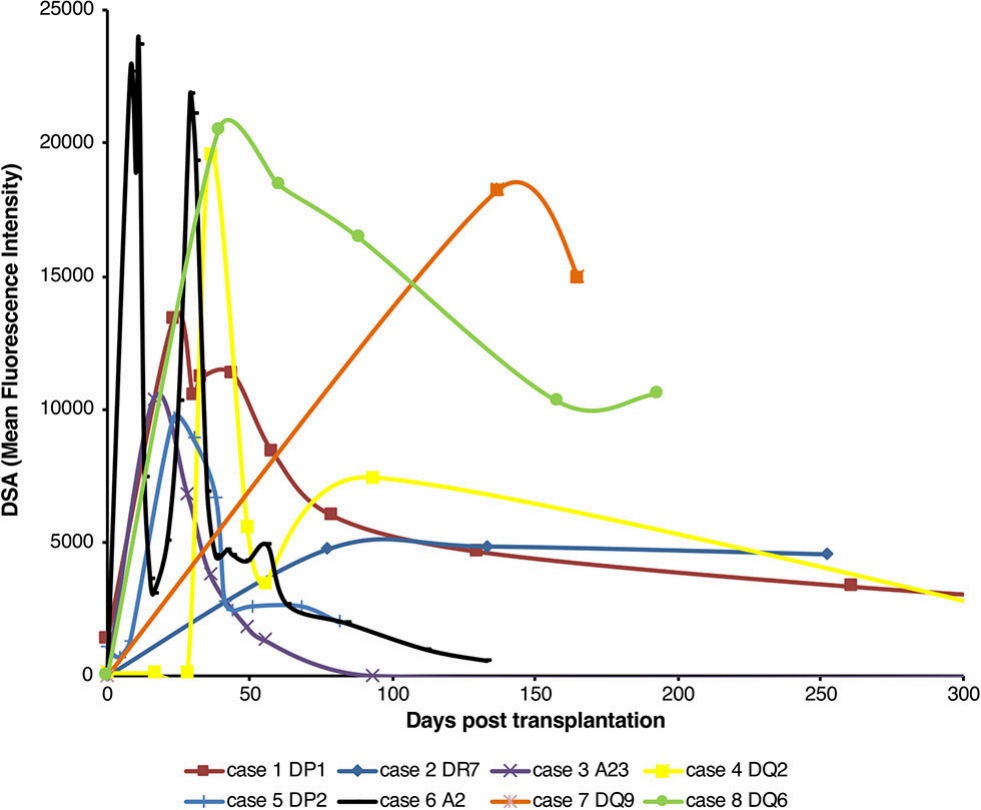
Pattern of *de novo* alloantibody development after kidney transplantation. The development of donor specific alloantibodies (DSA) against donor HLA antigen in eight human kidney transplant recipients (representative of a general cohort that were known to have DSA and whose alloantibody profile was serially assessed) was characterized by single-antigen HLA bead analysis of recipient sera. Recipient sera were analyzed for DSA until day 500 post-transplant; data is presented for the first 300 days, with cases 1 and 4 showing a mean fluorescence intensity of 733 at day 503 and 640 at day 386, respectively. Two general patterns of DSA formation were identified: very high levels of DSA which were relatively short-lived; and DSA levels which plateaued and remained at appreciable levels for a much longer period.

Analysis of the development of *de novo* donor specific alloantibody (DSA) post-transplantation in non-sensitized recipients of first time kidney allografts revealed two different patterns (Figure 1). Some recipients generated high levels of DSA, but that waned within the first thirty days, whereas in others, levels of DSA were sustained over many months. Because these patterns are compatible with short-lived extrafollicular foci vs. LLPC output from a GC reaction, we sought to establish an animal model to study how cellular events occurring within the allospecific B cell population relate to the pattern of DSA detected in human recipients.

### Precursor Frequency of the Helper T Cell Subset Is Rate Limiting for Early Alloantibody Production

**Figure 2:**
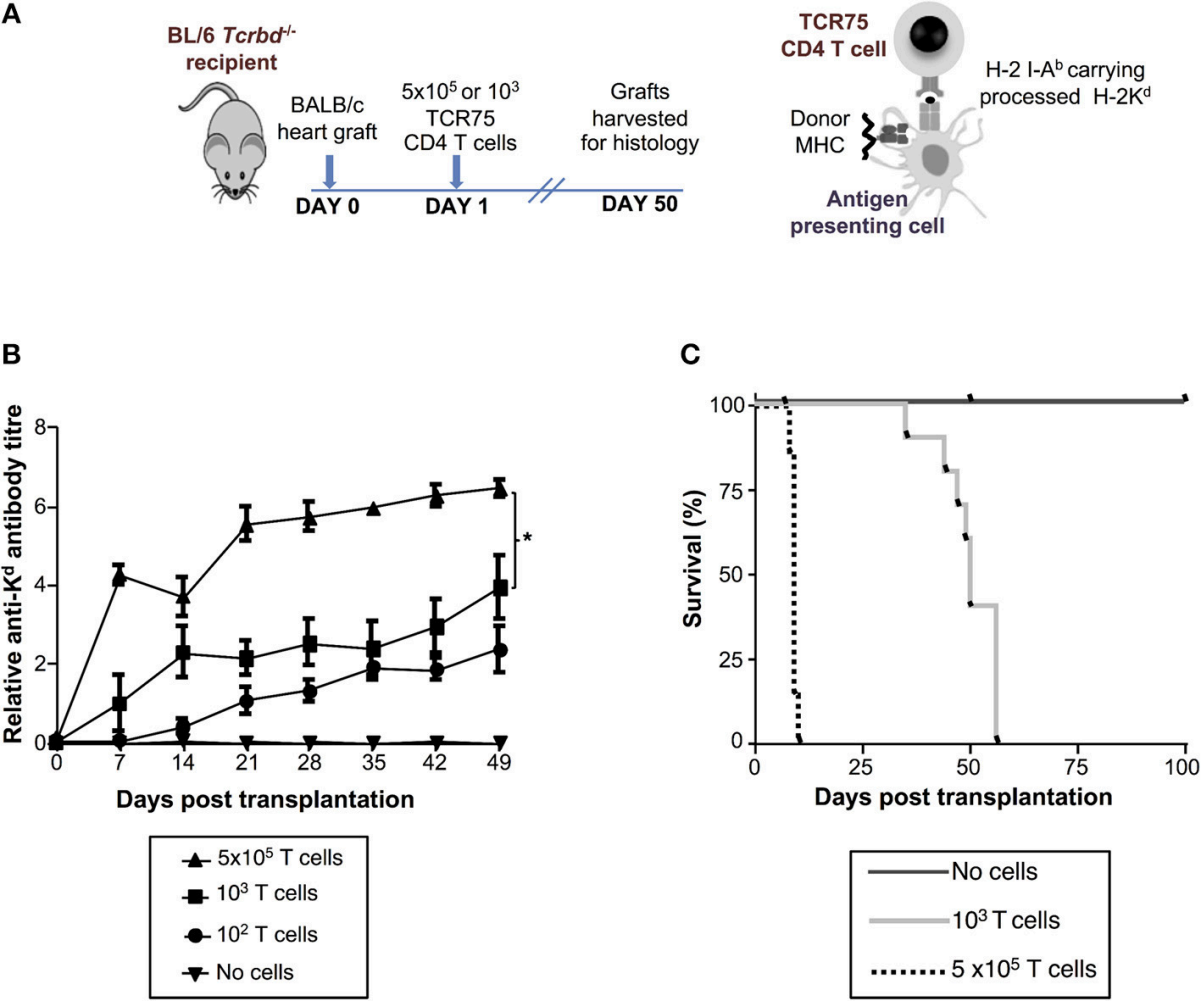
Development and characterization of murine model of antibody-mediated heart allograft rejection. **(A)** BL/6 *Tcrbd*^−/−^ recipients of BALB/c heart allografts were either unmodified (no cells group) or reconstituted the day after with 10^2^, 10^3^ (help-limited) or 5 × 10^5^ (help-unlimited) CD4 T cells from TCR transgenic *Rag1*^−/−^ TCR75 animals, which recognize I-A^b^ -restricted H-2K^d^_54−68_ peptide. **(B)** Development of anti-H-2K^d^ IgG antibody in reconstituted recipients (mean and S.E.M of *n* = 5 mice/group, ^*^P < 0.001 for 5 × 10^5^ vs. 10^3^; *p* = 0.08 for 10^3^ vs. 10^2^ T cells, two-way ANOVA). **(C)** Reconstitution with limiting numbers of TCR75 CD4 T cells resulted in gradual allograft failure (MST = 50 days; *n* = 10), whereas grafts rejected acutely in help-unlimited recipients (MST = 9 days, *P* < 0.001, log-rank test; *n* = 10). Unmodified BL/6 *Tcrbd*^−/−^ recipients that were not reconstituted (no cells, *n* = 6) did not reject the heart allograft.

We have previously reported that T cell receptor (TCR)-transgenic *Rag1*^−/−^ TCR75 CD4 T cells [that recognize I-A^b^-restricted H-2K${}_{54-68}^{\text{d}}$ allopeptide via the indirect pathway (26, 38)] can provide T helper cell function for humoral responses against the H-2K^d^ alloantigen of a BALB/c heart allograft (12, 13). We anticipated that because T cell help is a limiting determinant for early alloantibody production (39), the magnitude of the alloantibody response would be influenced by T helper cell availability. Thus, T cell-deficient *Tcrbd*^−/−^ mice were reconstituted with varying numbers of TCR75 CD4 T cells (10^2^, 10^3^, or 5 × 10^5^) the day after challenge with a BALB/c heart allograft (Figure 2A). As shown previously for other TCR transgenic CD4 T cell lines (12, 40), TCR75 CD4 T cells do not undergo discernible homeostatic proliferation when transferred into unchallenged *Tcrbd*^−/−^ mice (Figure S2). We therefore reasoned that expansion of the transferred TCR75 CD4 T cells would occur only in response to recognition of target antigen, and that the differences in numbers of transferred cells would be preserved, at least initially, in the recipients. We further reasoned that the different profiles of alloantibody generated according to the number of transferred TCR75 CD4 T cells would manifest as differences in the kinetics of allograft rejection, because there would be no opportunity for classical CD8 T cell-mediated cytotoxicity (41, 42). Similarly, whereas “direct-pathway” CD4 T cells can provoke rapid allograft rejection through cytotoxic destruction of allograft cells expressing MHC class II alloantigen (43), the relatively small numbers of indirect-pathway CD4 T cells that were transferred would be incapable of independently effecting acute heart graft rejection, because the allograft does not express target I-A^b^-restricted allopeptide epitope immediately after transplantation (44, 45). We have recently briefly described this model in relation to NK cell allorecognition of passenger lymphocytes (46).

Reconstitution of BL/6 *Tcrbd*^−/−^ mice with as few as 10^2^ TCR75 CD4 T cells provoked long-lasting, IgG humoral alloimmunity against H-2K^d^ alloantigen of the BALB/c heart graft (Figure 2B). A broadly similar response was observed following transfer of 10^3^ TCR75 CD4 T cells (henceforth termed the “help-limited” group). In comparison, the anti-K^d^ alloantibody response in recipient mice reconstituted with 5 × 10^5^ TCR75 CD4 T cells (henceforth the “help-unlimited” group) was markedly stronger at all time-points and developed more rapidly, such that appreciable levels of alloantibody were detectable 1 week after transplant (Figure 2B). The transferred TCR75 CD4 T cells mediated heart allograft rejection, because whereas BALB/c heart allografts survived indefinitely in unmodified BL/6 *Tcrbd*^−/−^ recipients, transfer of even small numbers (10^3^) of TCR75 CD4 T cells resulted in gradual graft failure (Figure 2C, median survival time (MST) = 50 days). Kinetics of heart graft rejection were, however, markedly different in the help-unlimited group, in that all grafts were rejected acutely with a MST of 9 days (Figure 2C).

### Histopathological Confirmation of Acute Humoral Rejection

**Figure 3:**
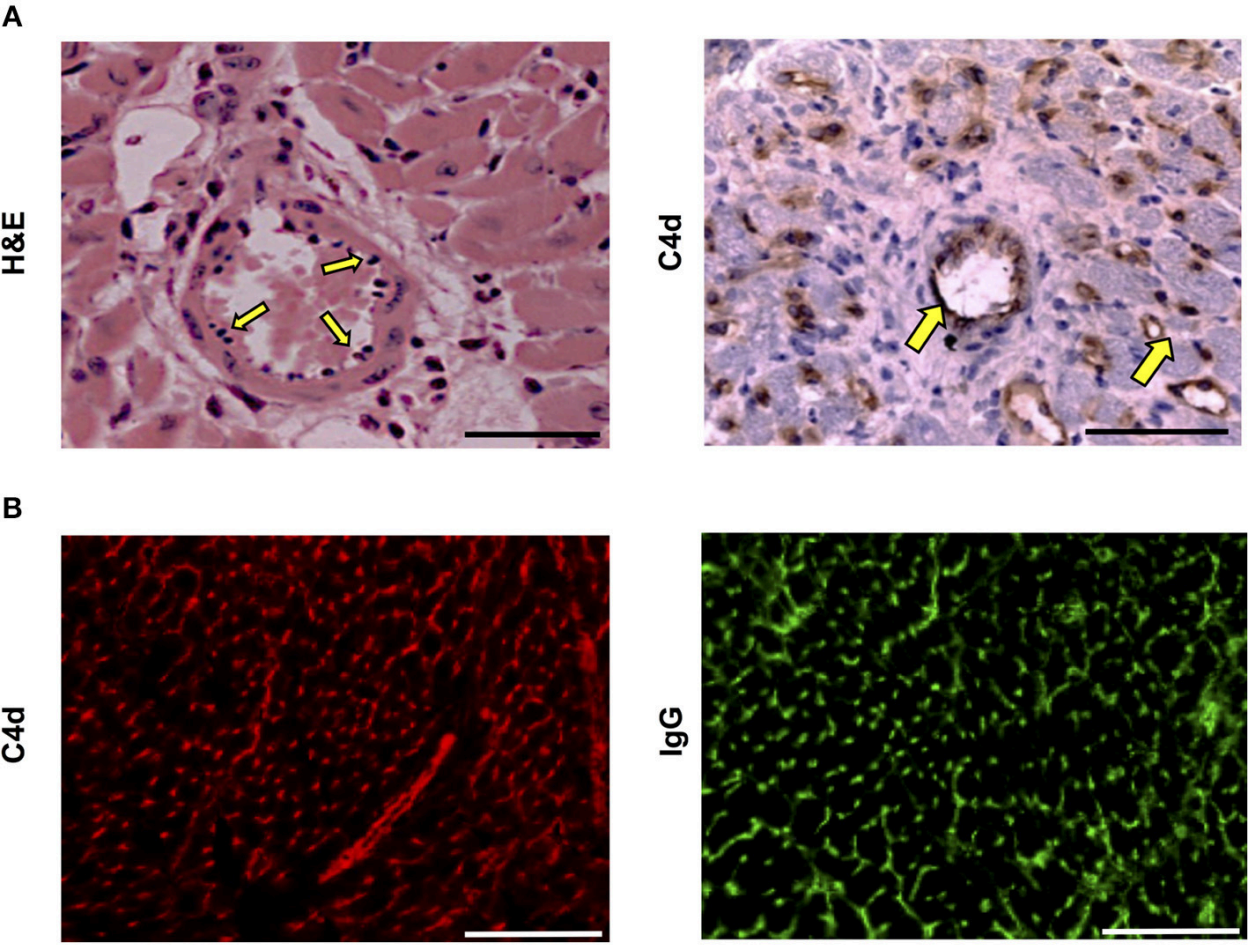
Histopathological confirmation of humoral rejection in help-unlimited recipients. **(A)** Day 6 BALB/c hearts explanted from BL/6 *Tcrbd*^−/−^ recipients reconstituted with 5 × 10^5^ TCR75 CD4 T cells demonstrated (*left*) widespread myocyte death (loss of striation), endothelial plumping and peri-vascular edema, and (*right, arrows*) strong endothelial complement C4d deposition. **(B)** Representative photomicrographs of immunofluorescence staining showing interstitial capillary staining for C4d (red, *left*); scale bar−50 μm and IgG deposition (green, *right*; scale bar−50 μm).

Histopathological examination of acutely rejecting BALB/c heart allografts explanted at day six from help-unlimited recipients revealed a striking absence of cellular rejection (Figure 3A). Instead there was widespread myocyte death, hemorrhage, edema, associated with “plumping” of the endothelium (Figure 3A, *left*). Immuno-histochemical labeling with anti-C4d antibody showed diffuse parenchymal staining (Figure 3A, *right*), possibly reflecting acute myocyte death, but in addition, there was strong and distinct endothelial complement deposition, associated with intragraft IgG deposition (Figure 3B). These features are thus consistent with severe (Grade 3) acute antibody-mediated rejection (47) as the principal cause of graft failure.

### Acute AMR Is Mediated by Extrafollicular Alloantibody Responses

**Figure 4:**
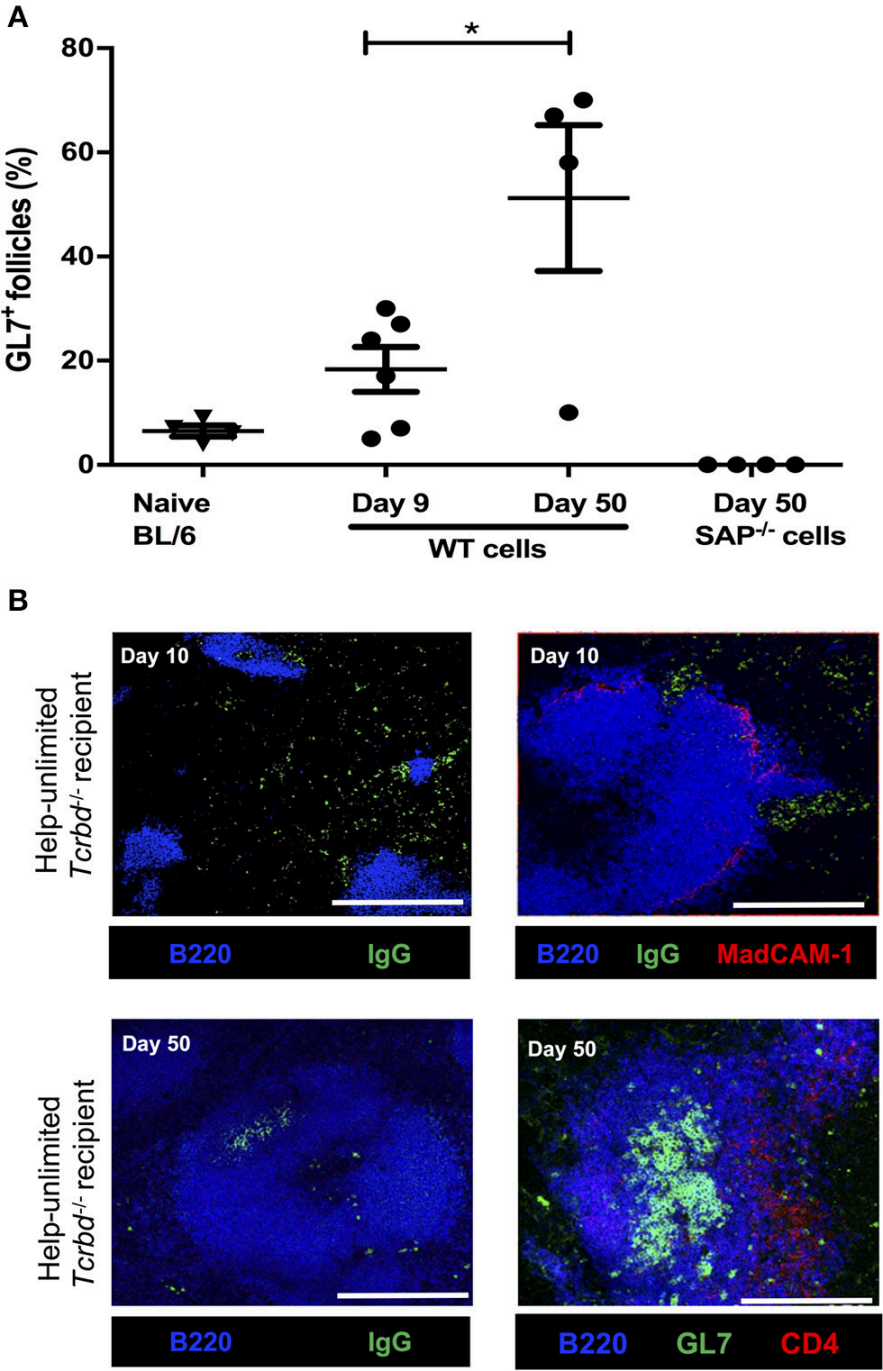
Extrafollicular and germinal center humoral responses during acute AMR. **(A)** Histogram of secondary (GL7^+ve^) splenic follicles expressed as percentage of total follicles within spleens of BL/6 *Tcrbd*^−/−^ recipients reconstituted with 5 × 10^5^ of WT or *Sh2d1a*^−/−^ (SAP^−/−^) TCR75 CD4 T cells. ^*^P = 0.03, two-tailed Student's *t*-test. **(B)** Representative confocal imaging of splenic sections of help-unlimited BL/6 *Tcrbd*^−/−^ recipients; at day 10 (top panel; scale bar−500 and 250 μm, respectively), IgG-switched B cells (green) are located predominantly in the extrafollicular space, close to the marginal sinus (MadCAM-1; red), whereas at day 50 (bottom panel; scale bar–250 μm), IgG switched cells and GL7^+^ GCs (green) are located within the follicle (B220; blue). Data represents mean ± S.E.M of a minimum of 5 animals/group, with each dot representing the biological replicate in a distinct group.

Although we have previously shown that transferred TCR75 CD4 T cells can provide T_FH_ cell function for generating GC responses against H-2K^d^ alloantigen (13), we thought it likely that the rapid rejection observed in the help unlimited group preceded development of the GC reaction. In support, splenic GC activity was barely above background by day 9 (the median time to graft rejection), but by day 50, approximately half the B cell follicles exhibited a GL7^+ve^ activated phenotype (Figure 4A). In addition, splenic confocal imaging confirmed that at day 10 in the help unlimited group, class-switched, IgG^pos^ (antibody-producing) cells were confined outside of the B220^pos^ B cell follicles, in extrafollicular foci close to the marginal sinus (Figure 4B). However, seven weeks after transplant, IgG^pos^ cells in the help-unlimited group were confined within B cell follicles (Figure 4B). Thus, these results suggest that the early alloantibody response observed in the help-unlimited group is predominantly a consequence of an extrafollicular response.

**Figure 5:**
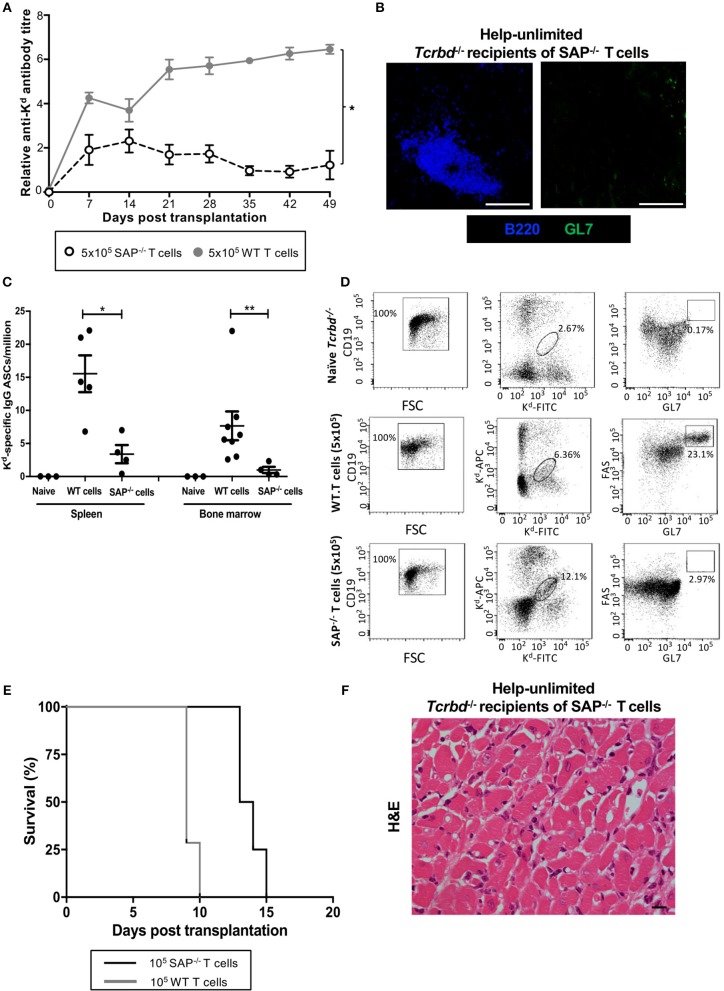
AMR is mediated by extrafollicular alloantibody responses in help-unlimited recipients. BL/6 *Tcrbd*^−/−^ recipients were reconstituted with 5 × 10^5^ wild-type (help-unlimited WT) TCR75 CD4 T cells (*n* = 7) or 5 × 10^5^ *Sh2d1a*^−/−^ (help unlimited SAP^−/−^) TCR75 CD4 T cells (*n* = 4) at challenge with a BALB/c heart allograft. **(A)** Compared with the WT group (copied from Figure 2B for ease of comparison), anti-H-2K^d^ IgG alloantibody responses in the help-unlimited SAP^−/−^ group were not sustained. ^*^P < 0.0001 two-way ANOVA. **(B)** Representative immunofluorescent staining of day 50 splenic sections from help-unlimited SAP^−/−^ and WT recipients, confirming an absence of GL7^+ve^ (green) GC activity in the SAP^−/−^ group: scale bar−250 μm. **(C)** ELISPOT assay of splenic and bone-marrow (BM) anti-K^d^ IgG antibody secreting cells (ASCs) 50 days after transplantation. Numbers of BM ASCs in recipients reconstituted with SAP^−/−^ TCR75 CD4 T cells were not above background; ^*^P = 0.03, ^**^P = 0.004, Mann-Whitney test. **(D)** Splenic H-2K^d^ -specific B cells were identified by flow cytometric detection of binding of CD19^+ve^ B cells to FITC-conjugated and APC-conjugated synthetic H-2K^d^ tetramers in naïve unchallenged BL/6 *Tcrbd*^−/−^, help-unlimited WT and help-unlimited SAP^−/−^ recipients, 6–7 weeks after challenge with a BALB/c graft. Gated cells in middle and right column of representative dot plots show percentage of enriched CD19^+ve^ B cells binding H-2K^d^ tetramer, and percentage of GC-specific (FAS^hi^GL7^pos^) tetramer bound CD19^+ve^ B cells, respectively. Whereas, transplantation provoked expansion of the H-2K^d^ specific B cell population in the help unlimited SAP^−/−^ recipients, GC B cells were not detectable. **(E)** BALB/c heart allografts were rejected acutely in the help-unlimited WT (*n* = 7, MST-9 days) and help unlimited SAP^−/−^ (*n* = 4, MST-13.5 days) recipient groups. (**F)** Day 10 BALB/c hearts explanted from help unlimited SAP^−/−^ recipients showing widespread myocyte death (loss of striation pattern), but an absence of cellular lymphocytic infiltration.

To confirm that acute humoral rejection could be mediated exclusively by an extrafollicular response, *Tcrbd*^−/−^ recipients of a BALB/c heart allograft were instead reconstituted with *Sh2d1a*^−/−^ TCR75 CD4 T cells. *Sh2d1a*^−/−^ T cells lack expression of SLAM-associated protein (SAP), which is essential for the prolonged physical interactions between B and T cells that leads to generation of the T_FH_ cell subset (19, 48–51). GC activity does not therefore occur, but *Sh2d1a*^−/−^ T cells can still provide help for extrafollicular responses. Recipients reconstituted with 5 × 10^5^ *Sh2d1a*^−/−^ TCR75 CD4 T cells (help-unlimited SAP^−/−^) generated robust early anti-K^d^ alloantibody responses, of a magnitude similar to that initially observed in the help-unlimited wild-type group (Figure 5A), but which waned thereafter, in keeping with the inability to form GCs responses (Figure 5B). The lack of the GC response was also evident in the absence of deposition of H-2K^d^ specific long-lived plasma cells (LLPCs) in the bone marrow (Figure 5C). Similarly, identification of H-2K^d^ allospecific B cells by labeling with synthetic H-2K^d^ tetramer revealed that, although the population expanded following challenge with a BALB/c heart allograft in the help-unlimited SAP^−/−^ recipients, acquisition of GL7^hi^FAS^hi^ GC surface phenotype did not occur (Figure 5D). Despite the absence of a GC response, the strong extrafollicular alloantibody response generated in the help-unlimited SAP^−/−^ recipients resulted in rapid rejection of BALB/c heart allografts (Figure 5E, MST 13.5 days), similar in tempo to that observed in the help-unlimited WT recipients (MST 9 days). The explanted heart allografts similarly exhibited characteristic features of acute humoral rejection, and an absence of lymphocytic infiltrates (Figure 5F).

### Alloantibody as an Effector Mechanism for Acute AMR

**Figure 6:**
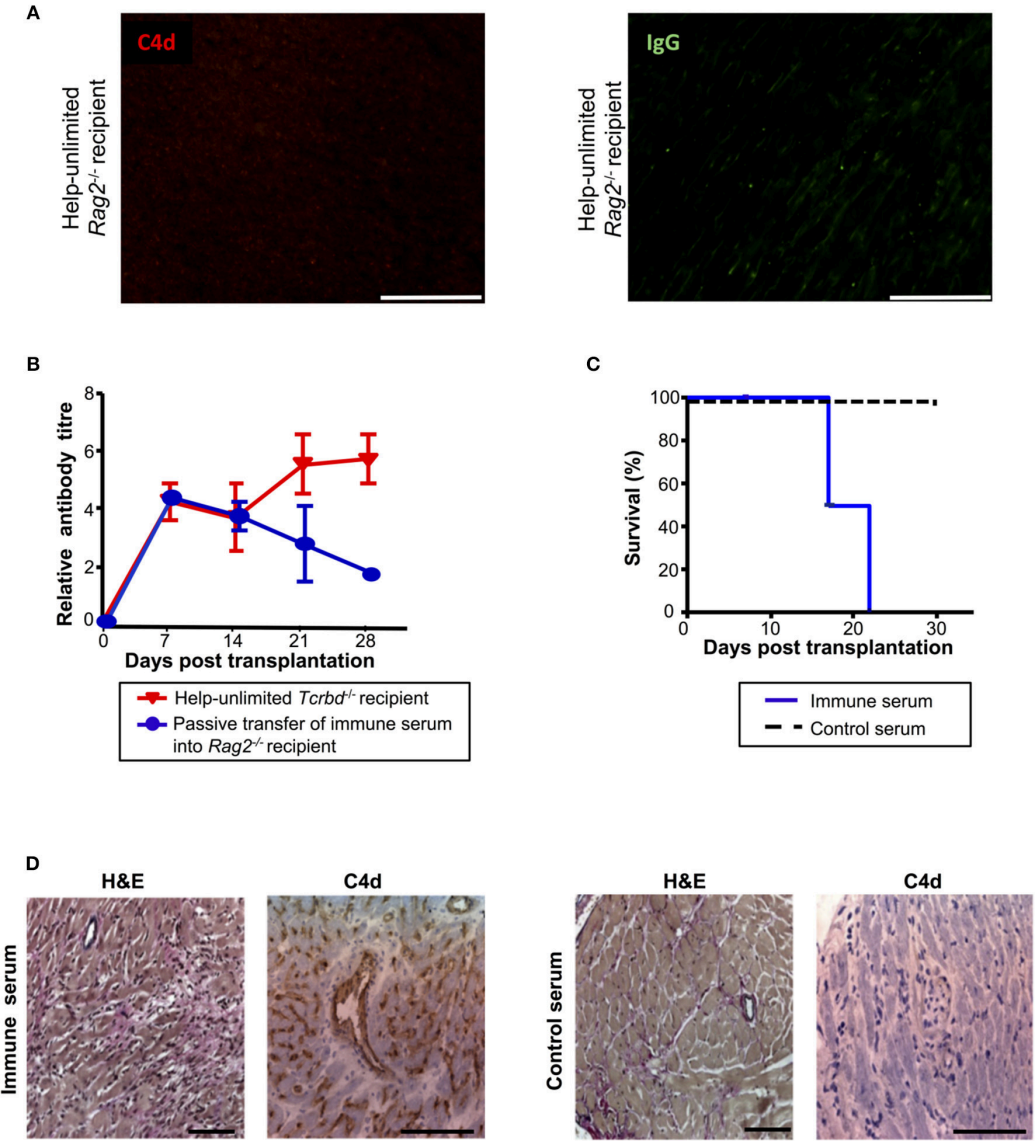
Alloantibody mediates endothelial activation and complement deposition. **(A)** Representative photomicrographs of immunofluorescence staining with no evidence of interstitial capillary staining for C4d (red, *left*) and IgG deposition (green, *right*; scale bars−50 μm) in BALB/c cardiac allografts explanted (at day 50) from *Rag2*^−/−^ recipients reconstituted with 5 × 10^5^ TCR75 CD4 T cells. **(B)** Anti-H-2K^d^ IgG antibody (mean ± S.D.) in *Rag2*^−/−^ recipients of BALB/c allografts injected with day 50 serum pooled from either help-unlimited BL/6 *Tcrbd*^−/−^ (immune serum, *n* = 5) or help-unlimited *Rag2*^−/−^ recipients (control serum, *n* = 5). **(C)** Transfer of immune serum led to acute graft loss, whereas transfer of control serum resulted in indefinite heart allograft survival (*P* < 0.001, log-rank test). **(D)** Histology of explanted hearts (day 7) demonstrated widespread myocyte damage and endothelial C4d deposition following transfer of immune serum (*left*), in comparison with normal histology and negative staining at day 30 following control serum transfer (*right*). Images are representative of 3 animals; scale bars−100 μm (H&E) and 150 μm (C4d).

Although the histological features of heart allografts in the acutely-rejecting, help-unlimited WT group are strongly suggestive of humoral rejection, we sought to confirm that alloantibody could independently effect graft damage. In this regard, BALB/c heart grafts survived indefinitely, without evidence of IgG or endothelial complement C4d deposition (Figure 6A), when transplanted into T and B cell deficient *Rag2*^−/−^ recipients, even when their T cell compartment was restored by adoptive transfer of 5 × 10^5^ WT TCR75 CD4 T cells. A direct effector role for alloantibody was then examined by passive transfer of immune serum (pooled from BL/6 *Tcrbd*^−/−^ recipients reconstituted with 5 × 10^5^ TCR75 CD4 T cells) into *Rag2*^−/−^ recipients of a BALB/c heart allograft. Passive transfer of immune serum achieved antibody titres in the *Rag2*^−/−^ recipients similar to those observed in the help-unlimited BL/6 *Tcrbd*^−/−^ recipients for the first 3 weeks (Figure 6B) and resulted in early graft rejection (Figure 6C). Rejecting grafts showed acute myocyte loss, oedema, endothelial swelling and invading neutrophils, along with extensive endothelial C4d deposition (Figure 6D, *left panel*). Injection of equivalent amounts of control serum from unmodified *Rag2*^−/−^ recipients of a BALB/c heart allograft did not prompt graft rejection (Figure 6C), and no graft damage was evident on histological examination (Figure 6D, *right panel*).

### The Relative Strength of the Extra-Follicular and GC Response Is Influenced by Precursor Proportions of Helper T Cells and Antigen Specific B Cells

**Figure 7:**
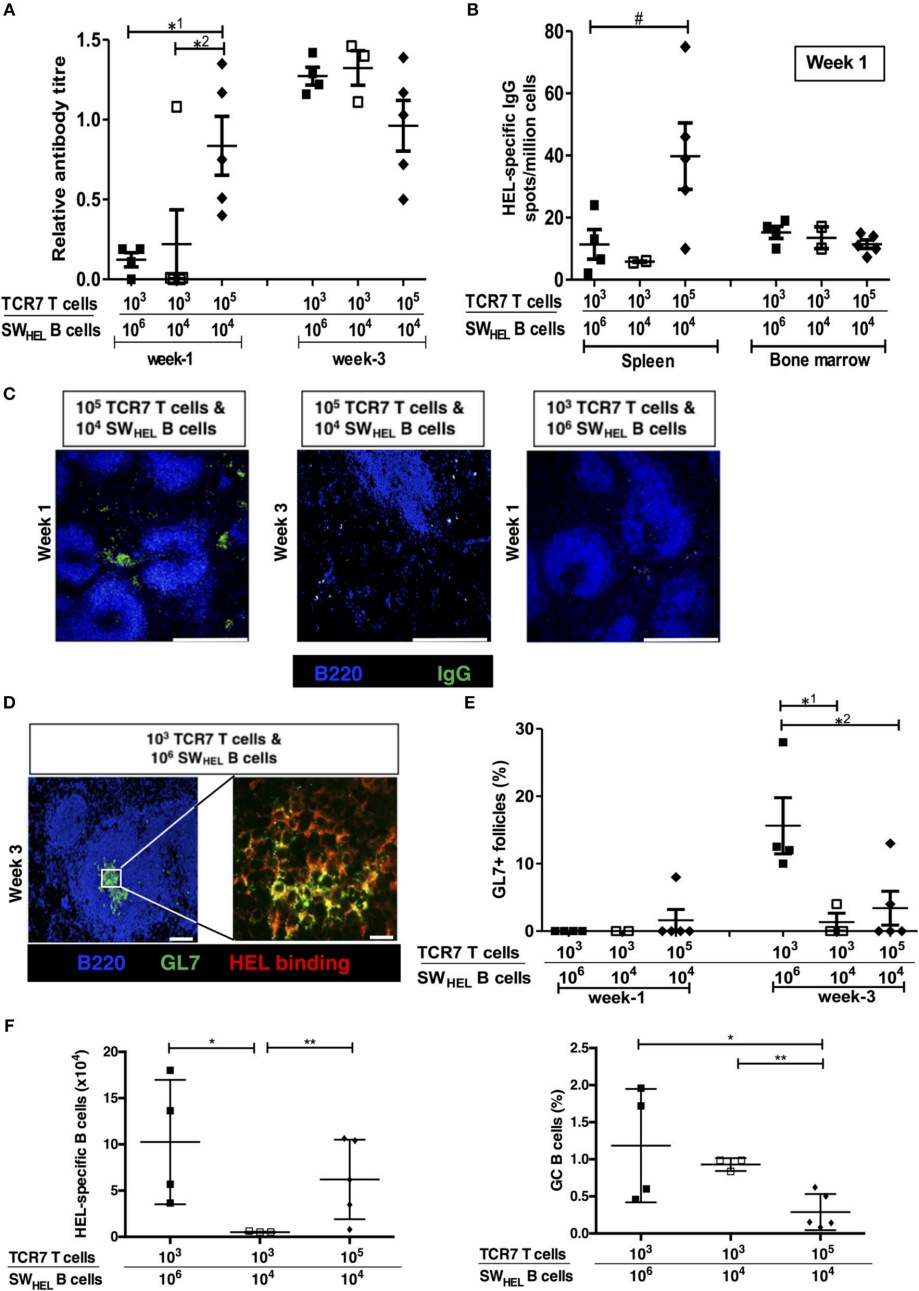
Numbers of antigen-specific B cells and helper T cells determine the size of the germinal center vs. extrafollicular response. Humoral responses in BL/6 *Tcrbd*^−/−^ mice immunized with Hen Egg Lysosyme (HEL) protein and adoptively transferred with different numbers of HEL-specific SW_HEL_ B cells and TCR7 CD4 T cells. **(A)** Anti-HEL IgG (mean ± S.E.M, with each dot representing the biological replicate in a distinct animal) measured 1 and 3 weeks after immunization; ^*^^1^*P* = 0.01, ^*^^2^*P* = 0.04 Mann-Whitney test. **(B)** ELISPOT assay of splenic and bone marrow anti-HEL IgG antibody-secreting cells revealed abundant numbers after 1 week in mice adoptively transferred with large (10^5^) numbers of TCR7 CD4 T cells; ^#^*P* = 0.06, Mann-Whitney test. **(C)** Representative confocal photomicrographs (scale bars-−500 μm) of immunofluorescence staining of splenic sections at 1 and 3 weeks after immunization, depicting B220 B cells (blue) and IgG positive (green) antibody secreting cells: numbers of adoptively transferred TCR7 T cells and SW_HEL_ B cells as indicated. IgG secreting cells are readily identified within extrafollicular foci at week 1 in mice transferred with large numbers (10^5^) of TCR7 CD4 T cells. **(D)** Representative confocal photomicrographs depicting HEL-specific germinal center activity (B220 B cell—Blue; GL7—green; HEL binding—red) at 3 weeks following HEL challenge in mice adoptively transferred with HEL-specific CD4 T and B cells, as indicated (*left*; scale bar−100 μm; *right*; scale bar-−20 μm). **(E)** Frequency of HEL-specific GL7^+^ GCs detected by immunofluorescence (expressed as percentage of total follicles within spleens of recipient BL/6 *Tcrbd*^−/−^ mice). Very few HEL-specific GCs were present at 1 week, but were clearly present by 3 weeks in the group that received proportionally greater numbers of HEL-specific B cells (and low numbers of HEL-specific CD4 T cells); ^*^^1^*P* = 0.04, ^*^^2^*P* = 0.03 Mann-Whitney test. **(F)** Flow cytometric enumeration (see Figure S3) of splenic HEL-specific B cells (*left histogram*) and percentage with GC (FAS^hi^ GL7^+ve^) phenotype (*right histogram*), 3 weeks after HEL-challenge in mice reconstituted with HEL-specific SW_HEL_ B cells and TCR7 CD4 T cells, as indicated. *Left histogram*, ^*^P = 0.05 and ^**^P = 0.41; *right histogram*, ^*^P = 0.09 and ^**^P = 0.003; two-tailed Student's *t*-test.

Despite the initially stronger alloantibody response observed in the help-unlimited recipient group, it was notable that germinal center activity at late time points in the help unlimited and help-limited groups was similar [see Figure 4A and companion paper (52) -Figure 3B], suggesting that whereas the availability of T cell help influences the ‘strength' of the extrafollicular response, other factors determine GC development. Given that competition for limiting availability of T_FH_ cells is critical for selection of high-affinity variants generated by SHM within the GC (21), we hypothesized that GC activity would instead be governed by precursor frequency of the responding, antigen-specific B cell population. To test this, varying numbers (10^3^ or 10^5^) of CD4 T cells purified from TCR7 mice (that recognize I-A^b^-restricted HEL peptide) plus B cells purified from SW_HEL_ mice (10^4^ or 10^6^) were adoptively transferred into BL/6 *Tcrbd*^−/−^ mice that were immunized with HEL protein. The HEL-specific extrafollicular and GC responses were analyzed at 1 and 3 weeks after immunization, as described previously (53). At week 1, strong anti-HEL IgG responses were only observed in mice adoptively transferred with large (10^5^) numbers of TCR7 CD4 T cells, despite transfer of relatively few (10^4^) HEL-specific SW_HEL_ B cells in this group (Figure 7A). Simultaneous ELISPOT analysis revealed that appreciable numbers of splenic HEL-specific antibody-secreting-cells were recovered only from this group and not, notably, in the group that had received greater numbers (10^6^) of SW_HEL_ B cells but fewer T cells (Figure 7B). Immunofluorescent analysis confirmed that at this time, IgG^pos^ secreting cells were located exclusively within extrafollicular foci, but in keeping with the anti-HEL titres, only in the group that had been reconstituted with greater numbers of TCR7 CD4 T cells (Figure 7C, *left*). In contrast, by week three, similar anti-HEL antibody responses were observed in all groups, irrespective of the number of TCR7 helper CD4 T cells transferred (Figure 7A, *right histogram*). By this stage, the extrafollicular foci had dissipated (Figure 7C, *middle*) and instead HEL-specific GC activity was detectable (Figure 7D), but most obviously in the group that received greater numbers of HEL-specific B cells [and relatively low numbers of HEL-specific T cells (Figures 7D,E)]. Flow cytometric analysis confirmed the presence of splenic HEL specific B cells that expressed a GC phenotype (Figures 7F, S3), and although this proportion appeared higher in the group that had received the larger number of HEL-specific B cells and relatively few HEL-specific CD4 T cells, numbers were too small to permit statistical comparison. In comparison, HEL-specific GC activity was lower in those mice that received greater numbers of HEL-specific CD4 T cells (Figure 7E), despite the robust early anti-HEL response observed in this group (Figure 7A). In the expectation that T_FH_ differentiation would be governed by the number of HEL-specific B cells, rather than precursor frequency of HEL-specific CD4 T cells, transferred TCR7 CD4 T cells that expressed CXCR5^hi^PD-1^hi^ T_FH_ phenotype were enumerated 3 weeks after challenge, but numbers recovered were too small to permit comparison. Nevertheless, these results support the hypothesis that the precursor frequency of the antigen-specific helper CD4 T cell population is a major factor in determining extrafollicular output, whereas GC activity is more strongly influenced by the starting population of antigen-specific B cells.

## Discussion

It has long been recognized that humoral immunity comprises several discrete phases, each providing distinctly different function. How these phases relate to the different clinicopathological manifestations of AMR is not known, and the increasing emphasis of the contribution of alloantibody to clinical transplantation suggests it is an important area for further study. Our findings that strong extrafollicular responses may mediate acute AMR, and that, conversely, GC responses are required for chronic AMR [see companion paper (52) and (54, 55)], therefore begin to provide some correlation between clinical events and the evolving dynamics of the allospecific B cell response.

Our conclusion that strong extrafollicular alloantibody responses can mediate rapid, acute rejection was confirmed experimentally by limiting T cell help to a population of allospecific helper CD4 T cells that lacked SAP expression. The histological features observed in acutely-rejecting allografts (endothelial plumping, neutrophil infiltration, complement deposition, and widespread myocyte death) resembled those that define acute AMR in human cardiac transplantation. Given the absence of direct-pathway cytotoxic T cell responses in our model (heart graft rejection was not observed in *Rag2*^−/−^recipients reconstituted with large numbers of WT CD4 T cells), our experiments therefore provide confirmation that such histological features are a direct consequence of alloantibody binding. Presumably, high titres of alloantibody produced by strong extrafollicular responses, despite being of relatively low affinity, can bind in sufficient concentration to allograft endothelium to trigger widespread complement activation and generation of the membrane attack complex. It should be noted, however, that in the recipient group reconstituted with wild-type TCR75 CD4 T cells (help-unlimited group), GC responses also developed, and it is possible that these also normally contribute to acute AMR.

Interestingly, transfer of large numbers of allospecific CD4 T cells was not associated, at least at late time points, with a more robust GC response than observed following transfer of lower numbers of CD4 T cells. Our experiments with the HEL system, in which it was possible to titer numbers of responding B cells and helper T cells, confirmed that the extrafollicular response was influenced profoundly by the availability of T cell help. At 1 week, the number of splenic IgG-secreting cells was determined by numbers of helper T cells (rather than antigen-specific B cells) that had been transferred, suggesting that increased availability of T cell help results in greater division of those B cells that are directed to the extrafollicular foci. This emphasis on an extrafollicular response is not due to lack of an established B cell follicular architecture in the T cell-deficient recipients at the time of challenge, because reconstitution with small numbers of antigen-specific T helper cells nevertheless polarized the subsequent B cell response strongly toward a germinal center reaction. A similar finding, but in relation to B cell receptor affinity, has been previously described (56). In contrast, GC activity was only minimally influenced by precursor frequency of the antigen-specific CD4 T cell population. Competition for limiting help from T_FH_ cells is critical for GC selection, and given that T_FH_ cell differentiation requires cognate B-T cell interaction (50), our experiments suggest that GC activity is mainly determined by the numbers, and response, of the antigen-specific B cell population; the antigen-specific helper T cell population simply plays a facultative role.

One potential concern is that our experimental findings are confounded by the homeostatic proliferation that potentially occurs upon transfer of the allospecific helper T cell population into T cell deficient mice. Against this, and in keeping with our previous studies (12, 13), transfer of a monoclonal population of TCR-transgenic TCR75 CD4 T cells into T cell deficient recipients did not result in substantial homeostatic proliferation. This has also been reported for non-transplant models (40). Nevertheless, we have previously shown that profound antigen-specific proliferation occurs in response to challenge with a K^d^-expressing heart allograft (13), and furthermore, that, in comparison to acute rejection, chronic rejection of BALB/c heart allografts is associated with a markedly expanded population (~5-fold) of chronically-dividing K^d^-peptide specific CD4 T cells (25). Thus, we anticipate that the chronic rejection observed in the help-limited group is associated with ongoing proliferation of the responding TCR75 CD4 T cell population, such that the relatively small number of T cells originally transferred has, by late time points, massively expanded and far exceeds the numbers administered in the help-unlimited group (that provoked acute allograft rejection). The critical difference in the humoral alloimmune response between the help-limited and unlimited groups is that in the first days after transplant, when initial T and B cell activation occurs, the much greater number of CD4 T cells in the help-unlimited group drives a strong extrafollicular response. In the help-limited group, by the time the transferred T cells have undergone an equivalent antigen-driven expansion, the B cell response is already geared toward a germinal center reaction.

Our finding that strong extrafollicular alloantibody responses are capable of independently mediating acute humoral rejection is likely to be clinically relevant. The transfer of large numbers of SAP-defective CD4 T cells resulted in an initially strong alloantibody response that effected acute heart graft rejection, but that thereafter waned, in the absence of a secondary GC response, according to the natural half-life of immunoglobulin. This may parallel the decay in alloantibody described in a cohort of patients whose acute AMR is successfully treated (57). Longer-term transplant outcomes for this cohort are acceptable, suggesting that modulation of the humoral alloimmune response to prevent progression from the extrafollicular to the GC stage may be critical in preventing development of chronic AMR. Finally, our description that extrafollicular and GC output is strongly influenced by the relative proportions of antigen-specific B and T lymphocytes suggests that particularly strong extrafollicular responses that mediate acute AMR may develop even when very few allospecific B cells are initially present, because an abundance of allospecific helper CD4 T cells would drive marked expansion and plasmablast transformation within the extrafollicular foci. This could conceivably occur when allospecific CD4 T cell memory responses have been established prior to the transplant (58, 59), possibly against additional alloantigens expressed on the graft other than those that are targeted by the allospecific B cell response (12). In adult human transplant recipients, the alloresponse is thought to consist principally of recall memory responses, often because of cross-reactive heterologous immunity (60, 61). Our ongoing murine experiments are examining whether stronger extrafollicular alloantibody responses against one alloantigen develop when help from recall memory CD4 T cells against additional “accessory” alloantigens is also available.

## Author Contributions

All authors contributed extensively to the work presented in this paper. MC and JA jointly conceived the study with RM and GP, designed and implemented the cardiac allograft rejection model with contributions from MQ, MM, JMA, and IG. ML contributed to characterization of germinal center responses. SP carried out analysis of DSA levels after kidney transplants. EM and MG performed cardiac allograft histopathological characterization. GP and RM prepared the manuscript, with contributions from MC, JA, VK, and ML.

### Conflict of Interest Statement

The authors declare that the research was conducted in the absence of any commercial or financial relationships that could be construed as a potential conflict of interest.

## Supplementary Material

Figure S1 Calculation of relative antibody serum titres. Typical absorbance versus dilution curves obtained from anti-H-2K^d^ IgG ELISA assay of serum samples (left) were subjected to area under the curve analysis and antibody levels compared to value obtained for control hyperimmune serum (positive control) (right histogram).

Figure S2 Absence of homeostatic proliferation of adoptively transferred TCR75 T cells in T cell deficient mice. Representative flow cytometry histogram (of three independent experiments), gating on CD4^+^, Thy1.1^+^, live (7-AAD^−^), CFSE-labeled 5 × 10^5^ TCR75 CD4 T cells, three days after adoptive transfer into BL/6 *Tcrbd*^−/−^ mice.

Figure S3 Flow cytometric analysis of HEL-specific B and CD4 T cells. Flow cytometric analysis of week 3 spleens for phenotypic characterization of B and T cells in BL/6 *Tcrbd*^−/−^ mice immunized with HEL protein and adoptively transferred with different numbers of HEL-specific SW_HEL_ B cells and TCR7 CD4 T cells. Lymphocytes were plotted against B cell (B220) and T cell (CD4) markers. A HEL-binding gate was applied on the B cell population prior to quantifying GC B cells (based on GL7 and FAS expression).

Table S1 Key resources table.
