# Supplementary figures and images for: Relative Frequencies of Alloantigen-Specific Helper CD4 T Cells and B Cells Determine Mode of Antibody-Mediated Allograft Rejection
Source: Front Immunol. 2019 Jan 22;9:3039. doi: 10.3389/fimmu.2018.03039 (PMC6357941; doi:10.3389/fimmu.2018.03039)

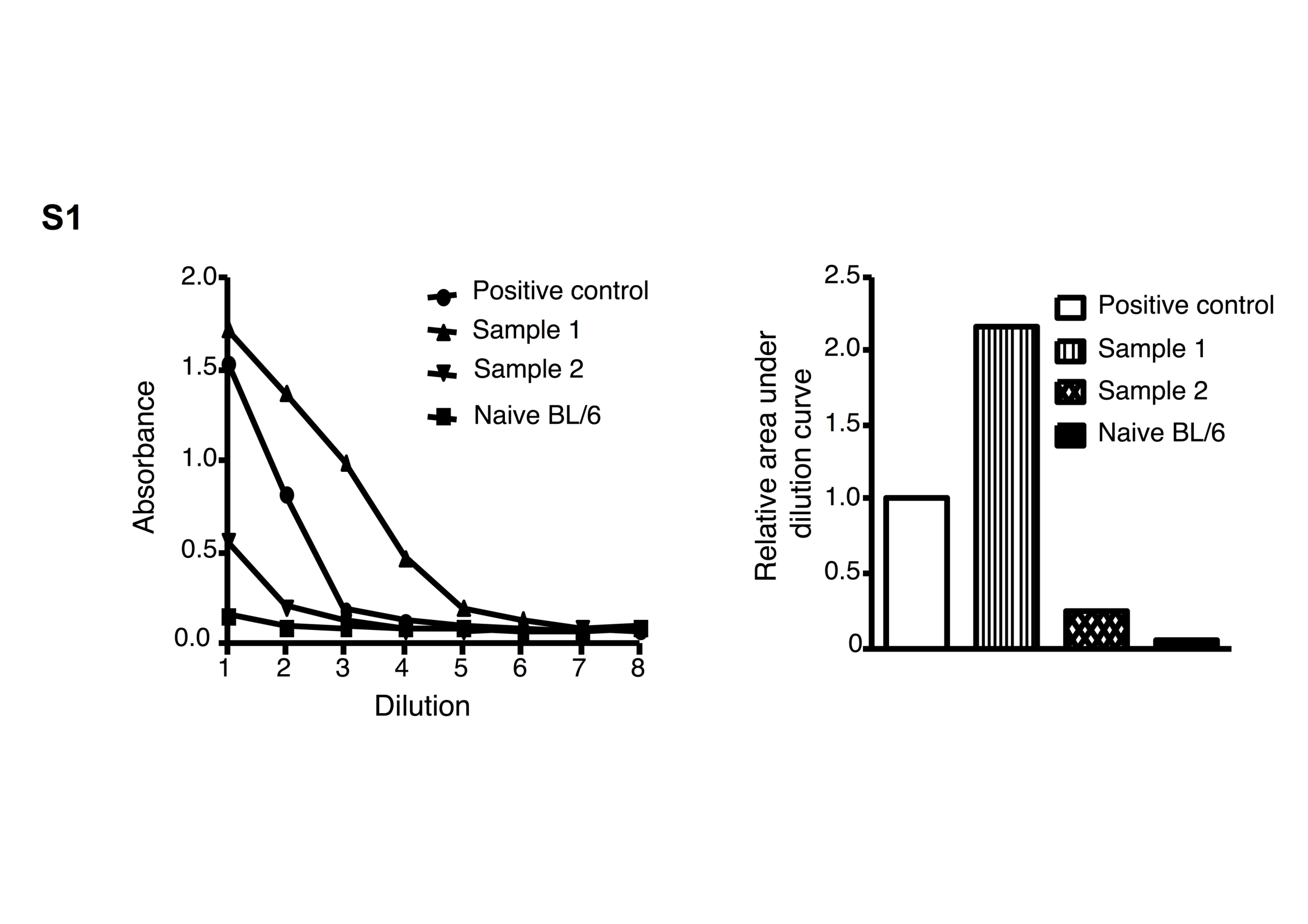

Supplement: Figure S1 — Calculation of relative antibody serum titres. Typical absorbance versus dilution curves obtained from anti-H-2Kd IgG ELISA assay of serum samples (left) were subjected to area under the curve analysis and antibody levels compared to value obtained for control hyperimmune serum (positive control) (right histogram). [file Image_1.TIFF]

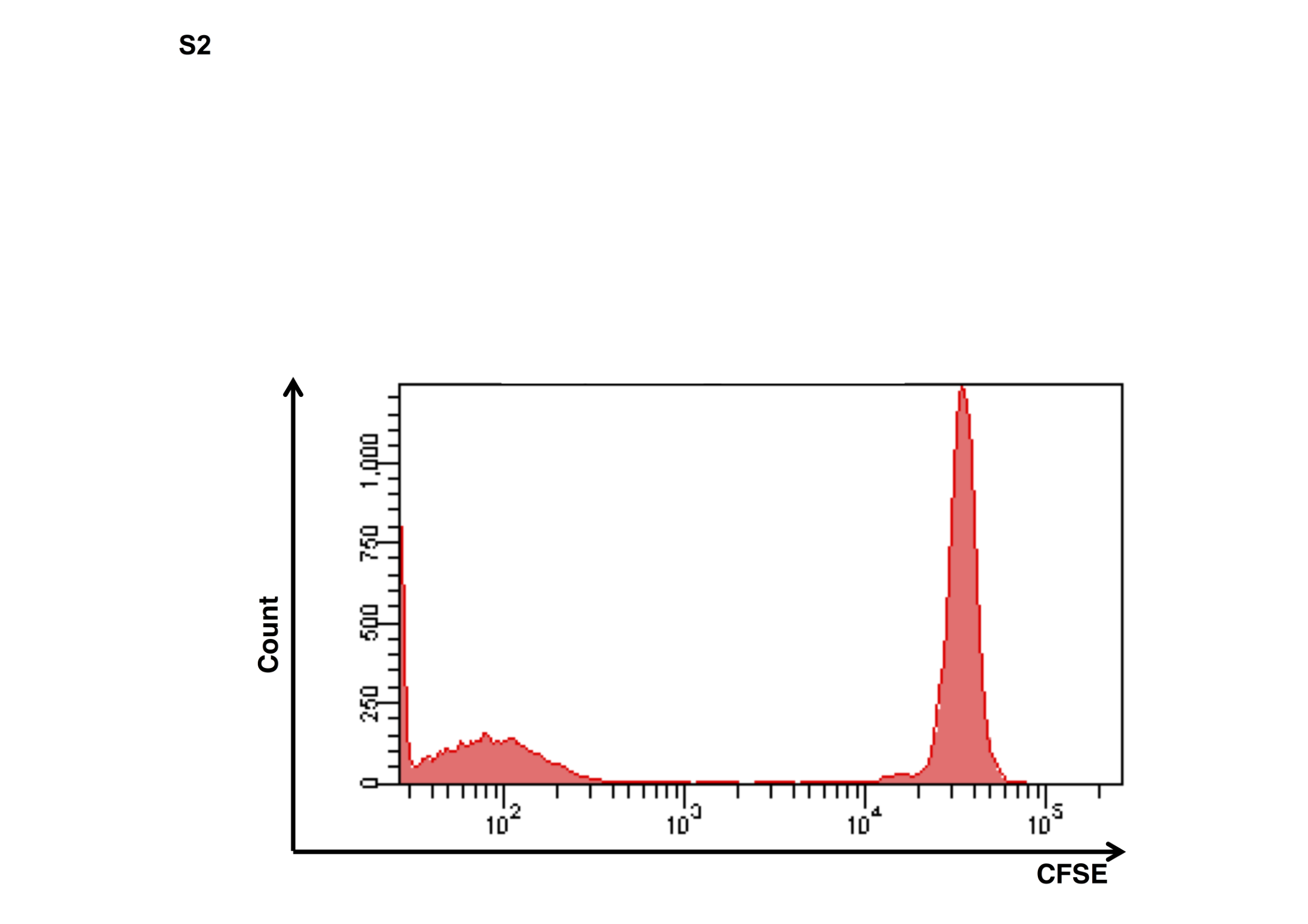

Supplement: Figure S2 — Absence of homeostatic proliferation of adoptively transferred TCR75 T cells in T cell deficient mice. Representative flow cytometry histogram (of three independent experiments), gating on CD4+, Thy1.1+, live (7-AAD−), CFSE-labeled 5 × 10^5^ TCR75 CD4 T cells, three days after adoptive transfer into BL/6 Tcrbd−/− mice. [file Image_2.TIFF]

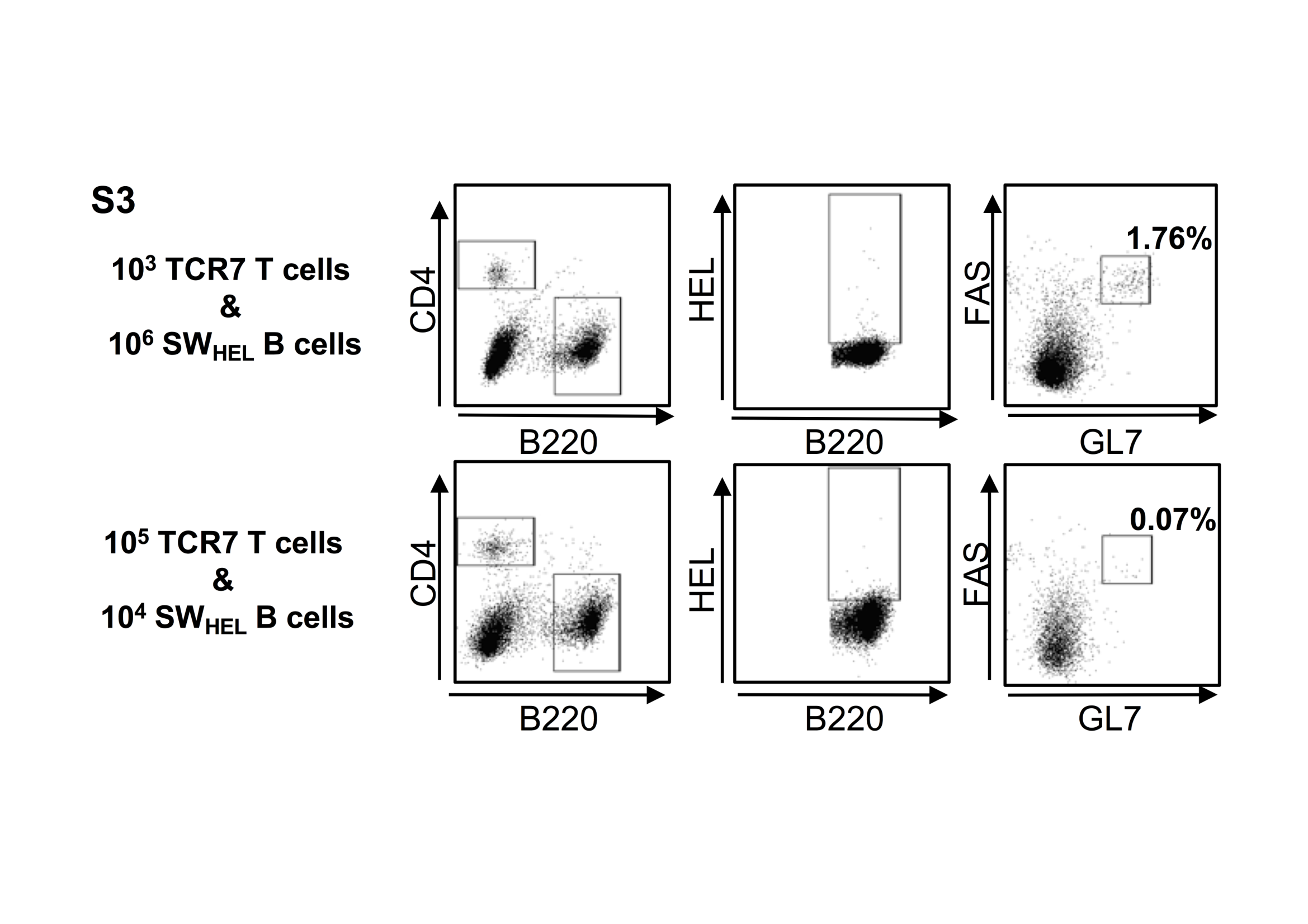

Supplement: Figure S3 — Flow cytometric analysis of HEL-specific B and CD4 T cells. Flow cytometric analysis of week 3 spleens for phenotypic characterization of B and T cells in BL/6 Tcrbd−/− mice immunized with HEL protein and adoptively transferred with different numbers of HEL-specific SWHEL B cells and TCR7 CD4 T cells. Lymphocytes were plotted against B cell (B220) and T cell (CD4) markers. A HEL-binding gate was applied on the B cell population prior to quantifying GC B cells (based on GL7 and FAS expression). [file Image_3.TIFF]
