# Supplementary material for: Relative Frequencies of Alloantigen-Specific Helper CD4 T Cells and B Cells Determine Mode of Antibody-Mediated Allograft Rejection
Source: Front Immunol. 2019 Jan 22;9:3039. doi: 10.3389/fimmu.2018.03039 (PMC6357941; doi:10.3389/fimmu.2018.03039)
Supplement: Table S1 — Key resources table. [file Table_1.docx]

***Table S1. Key Resources Table (paper 1)***

| **Reagent type (species) or resource** | **Designation** | **Source or Reference** | **Identifiers** | **Additional Information** |
| --- | --- | --- | --- | --- |
| Strain, strain background (*Mus musculus*) | C57BL/6 | Charles River Laboratories | Strain code 027 |  |
| Strain, strain background (*Mus musculus*) | Balb/c | Charles River Laboratories | Strain code 028 |  |
| Strain, strain background (*Mus musculus*) | BL/6.129P2-*Tcrb^tm1Mom^Tcrd^tm1Mom^*/J | Jackson Laboratory | Stock #2122 |  |
| Strain, strain background (*Mus musculus*) | C57BL/6 Rag2^-/-^ | Prof T. Rabbitts (Laboratory of Molecular Biology, Cambridge, UK) |  |  |
| Strain, strain background (*Mus musculus*) | TCR-transgenic Rag1^-/-^ TCR75 mice (H-2b), specific for I-A^b^–restricted H-2K^d^ 54–68 peptide | Prof. P. Bucy (University of Alabama, Birmingham, AL, USA) |  | (1) |
| Strain, strain background (*Mus musculus*) | BCR-transgenic SW_HEL_ (VH10_tar_^+/-^ x LC2) mice (H-2^b^) | Prof R. Brink (Garvan Institute of Medical Research, Darlinghusrt, Australia) |  | (2) |
| Strain, strain background (*Mus musculus*) | Sh2d1a^−/−^ | Dr S. Crotty (University of California, La Jolla, California) |  | (3) |
| Strain, strain background (*Mus musculus*) | TCR7 | Dr M Linterman (Laboratory of Lymphocyte Signalling and Development, Babraham Institute, Cambridge, UK) |  | (4)  TCR recognises the subdominant HEL I-A^b^ epitope HEL_74–88_ |
| Biological sample | Serum | Transplanted or naïve mice described in this paper |  |  |
| Antibody | anti-C4d | Abcam | 16-D2 |  |
| Antibody | IgG-FITC | BD Biosciences | G18-145 |  |
| Antibody | rat anti-mouse B220-APC | BD Biosciences | RA3-6B2 |  |
| Antibody | rat anti mouse GL7-FITC | BD Biosciences | clone GL7 |  |
| Antibody | Streptavidin-Alexa Fluor 555 | Thermo Fisher Scientific | S32355 |  |
| Antibody | rat anti-mouse CD4-biotin | BD Biosciences | H129.19 |  |
| Antibody | anti-CD19-PerCp | Miltenyi Biotec | clone LT19 |  |
| Antibody | HyHEL-10 | Absolute Antibody | cat# Ab00412-1.1 |  |
| Antibody | GL7-PE | BioLegend | clone GL7 |  |
| Antibody | FAS-PE-CY7 | BD Biosciences | clone Jo2 |  |
| Antibody | rat anti mouse-B220-FITC | BD Biosciences | clone RA3-6B2 |  |
| Antibody | Streptavidin-APC | Thermo Fisher Scientific | SA1005 |  |
| Peptide, recombinant protein | MHC class I tetramer (H-2K (d) Lm Llo 91-99GYKDGNEYI-APC | NIH Core Tetramer facility, Atlanta, GA, USA |  |  |
| Peptide, recombinant protein | MHC class I tetramer (H-2K (d) Lm Llo 91-99GYKDGNEYI-FITC | NIH Core Tetramer facility, Atlanta, GA, USA |  |  |
| Commercial assay or kit | Antibody Purification Kit | Thermo Scientific | cat# 89953 |  |
| Commercial assay or kit | Avidin/biotin blocking kit | Vector labs | cat# SP-2001 |  |
| Commercial assay or kit | Protein Quantification Kit-Rapid | Sigma-Aldrich | cat# 51254 |  |
| Chemical compound, drug | H-2K (d) protein | This paper |  |  |
| Chemical compound, drug | Hen egg lysozyme | Sigma-Aldrich | cat# L6876 |  |
| Chemical compound, drug | CellLytic M | Sigma-Aldrich | cat# C2978 |  |
| Software, algorithm | ForteBio (software 7.0.1.5) | <https://shop.fortebio.com/site-license-octet-data-analysis-software-version-7.x.html> |  |  |
| Software, algorithm | GraphPad vers. 4 | <https://www.graphpad.com/Downloads/InstallPrism403Updater.exe> |  |  |
| Software, algorithm | Flowjo 10.4.2 | https://s3-us-west-2.amazonaws.com/fjinstallers/FlowJo-Win64-10.4.2.exe |  |  |

1. K. Honjo, X. Yan Xu, J. A. Kapp and R. P. Bucy: Evidence for cooperativity in the rejection of cardiac grafts mediated by CD4 TCR Tg T cells specific for a defined allopeptide. *Am J Transplant*, 4(11), 1762-8 (2004)

2. T. G. Phan, M. Amesbury, S. Gardam, J. Crosbie, J. Hasbold, P. D. Hodgkin, A. Basten and R. Brink: B cell receptor-independent stimuli trigger immunoglobulin (Ig) class switch recombination and production of IgG autoantibodies by anergic self-reactive B cells. *J Exp Med*, 197(7), 845-60 (2003)

3. M. J. Czar, E. N. Kersh, L. A. Mijares, G. Lanier, J. Lewis, G. Yap, A. Chen, A. Sher, C. S. Duckett, R. Ahmed and P. L. Schwartzberg: Altered lymphocyte responses and cytokine production in mice deficient in the X-linked lymphoproliferative disease gene SH2D1A/DSHP/SAP. *Proc Natl Acad Sci U S A*, 98(13), 7449-54 (2001) doi:10.1073/pnas.131193098

4. M. Neighbors, S. B. Hartley, X. Xu, A. G. Castro, D. M. Bouley and A. O'Garra: Breakpoints in immunoregulation required for Th1 cells to induce diabetes. *Eur J Immunol*, 36(9), 2315-23 (2006) doi:10.1002/eji.200636432
